# Different loneliness types, cognitive function, and brain structure in midlife: Findings from the Framingham Heart Study

**DOI:** 10.1016/j.eclinm.2022.101643

**Published:** 2022-09-06

**Authors:** Qiushan Tao, Samia C. Akhter-Khan, Ting Fang Alvin Ang, Charles DeCarli, Michael L. Alosco, Jesse Mez, Ronald Killiany, Sherral Devine, Ami Rokach, Indira Swetha Itchapurapu, Xiaoling Zhang, Kathryn L. Lunetta, David C. Steffens, Lindsay A. Farrer, Douglas N. Greve, Rhoda Au, Wei Qiao Qiu

**Affiliations:** a Department of Pharmacology & Experimental Therapeutics, Boston University School of Medicine, Boston, MA, USA; b Framingham Heart Study, Boston University School of Medicine, USA; c Department of Health Service & Population Research, Institute of Psychiatry, Psychology & Neuroscience, King's College London, London, UK; d Department of Anatomy & Neurobiology, Boston University School of Medicine, Boston, MA, USA; e Department of Epidemiology, Boston University School of Public Health, Boston, MA, USA; f Slone Epidemiology Center, Boston University School of Medicine, USA; g Alzheimer's Disease Center, University of California Davis Medical Center, CA, USA; h Department of Neurology, Boston University School of Medicine, Boston, MA, USA; i Alzheimer's Diesease and Chronic Traumatic Encephalopathy Research Centers, Boston University, Boston, MA, USA; j Department of Psychiatry, Boston University School of Medicine, USA; k Department of Psychology, York University, Toronto, Canada; l Department of Medicine (Biomedical Genetics), Boston University School of Medicine, Boston, MA, USA; m Department of Biostatistics, Boston University School of Medicine, USA; n Department of Psychiatry, University of Connecticut School of Medicine, USA; o Martinos Center for Biomedical Imaging, Massachusetts General Hospital, Harvard University School of Medicine, USA

**Keywords:** Social isolation, Dementia, Alzheimer's disease, Neuroimaging, Cohort study, Brain health

## Abstract

**Background:**

It remains unclear whether persistent loneliness is related to brain structures that are associated with cognitive decline and development of Alzheimer's disease (AD). This study aimed to investigate the relationships between different loneliness types, cognitive functioning, and regional brain volumes.

**Methods:**

Loneliness was measured longitudinally, using the item from the Center for Epidemiologic Studies Depression Scale in the Framingham Heart Study, Generation 3, with participants’ average age of 46·3 ± 8·6 years. Robust regression models tested the association between different loneliness types with longitudinal neuropsychological performance (*n =* 2,609) and regional magnetic resonance imaging brain data (*n =* 1,829) (2002-2019). Results were stratified for sex, depression, and Apolipoprotein E4 (*ApoE*4).

**Findings:**

Persistent loneliness, but not transient loneliness, was strongly associated with cognitive decline, especially memory and executive function. Persistent loneliness was negatively associated with temporal lobe volume (*β* = −0.18, 95%CI [−0.32, −0.04], *P* = 0·01). Among women, persistent loneliness was associated with smaller frontal lobe (*β* = −0.19, 95%CI [−0.38, −0.01], *P* = 0·04), temporal lobe (*β* = −0.20, 95%CI [−0.37, −0.03], *P* = 0·02), and hippocampus volumes (*β* = −0.23, 95%CI [−0.40, −0.06], *P* = 0·007), and larger lateral ventricle volume (*β* = 0.15, 95%CI [0.02, 0.28], *P* = 0·03). The higher cumulative loneliness scores across three exams, the smaller parietal, temporal, and hippocampus volumes and larger lateral ventricle were evident, especially in the presence of *ApoE*4.

**Interpretation:**

Persistent loneliness in midlife was associated with atrophy in brain regions responsible for memory and executive dysfunction. Interventions to reduce the chronicity of loneliness may mitigate the risk of age-related cognitive decline and AD.

**Funding:**

US National Institute on Aging.

**Research in context**

**Evidence before this study**

The authors reviewed the literature using traditional sources (e.g., PubMed). Although the literature shows an association between loneliness and cognition, none of the studies distinguished between different types or chronicity of loneliness, such as persistent and transient loneliness, when investigating underlying brain volumes. Our previous research found that persistent loneliness, but not transient or incident loneliness, was a strong risk factor for the development of Alzheimer's disease (AD). However, the association between different loneliness types, longitudinal cognitive changes, and brain volumes in midlife remains unclear.

**Added value of this study**

Using data from the Framingham Heart Study (FHS) Generation 3, we found that persistent loneliness in midlife was more strongly associated with cognitive decline and brain atrophy than transient or incident loneliness. Having persistent feelings of loneliness was a strong predictor for cognitive decline and brain atrophy, specifically among women and *ApoE*4 carriers.

**Implications of all the available evidence**

People experiencing persistent loneliness in midlife are at higher risk of cognitive decline, brain atrophy, and dementia development, compared to people who recover from loneliness. Our study highlights the need for effective treatment to prevent chronic loneliness, and special attention should be paid to women and genetic risk carriers for AD.

Alt-text: Unlabelled box

## Introduction

Loneliness is a subjective experience—with cognitive, emotional, and behavioral dimensions—resulting from a discrepancy between desired and actual social relationships.^1^ Feeling lonely in midlife has been identified as a risk-factor for negative health outcomes including cardiovascular disease (CVD), stroke,^2^ and mortality,^3^ posing a threat to healthy aging. Whereas some people can recover from loneliness by applying various coping techniques,^4^ others suffer from persistent loneliness. In our recent publication, using data from the Framingham Heart Study's (FHS) second generation, we found that loneliness increases the risk for the development of Alzheimer's disease (AD) only when adults feel lonely persistently, not when loneliness is short-term.^5^ Consistently, another study found chronic loneliness to have stronger negative effects on cognitive function, evaluated by a single cognitive test (the Mini-mental State Exam (MMSE)), than transient loneliness.^6^ Thus, it seems reasonable to speculate that there may be different biological pathways linking loneliness types to cognitive functioning and brain structures, depending on the persistency of loneliness. Yet, the relationships between transient versus persistent loneliness with brain structures and cognitive changes remain unclear.

People who feel lonely persistently may not be as engaged in cognitively stimulating environments, which may result in detrimental effects on brain health. On the other hand, people who can recover from loneliness may likely be involved in more diverse activities and have more stimulating social interactions to prevent cognitive decline. The cognitive reserve hypothesis suggests that stimulating cognitive engagement is associated with better memory and executive functioning.^7^ It has been shown that both persistent sad mood and loneliness may be associated with areas of the default network of the brain.^8^^,^^9^ Therefore, we hypothesize that persistent but not transient loneliness is associated with cognitive decline and atrophy of brain regions related to AD pathology.

To examine the relationship between different loneliness types, cognitive function and brain volumes, we used data from the third generation of the FHS to define four loneliness types, i.e., no, incident, transient, and persistent loneliness, across two health exams.^5^ The purpose of the present study is to assess the association between different loneliness types in midlife with longitudinal cognitive changes and brain volumes.

## Methods

### Participants

**Figure 1:**
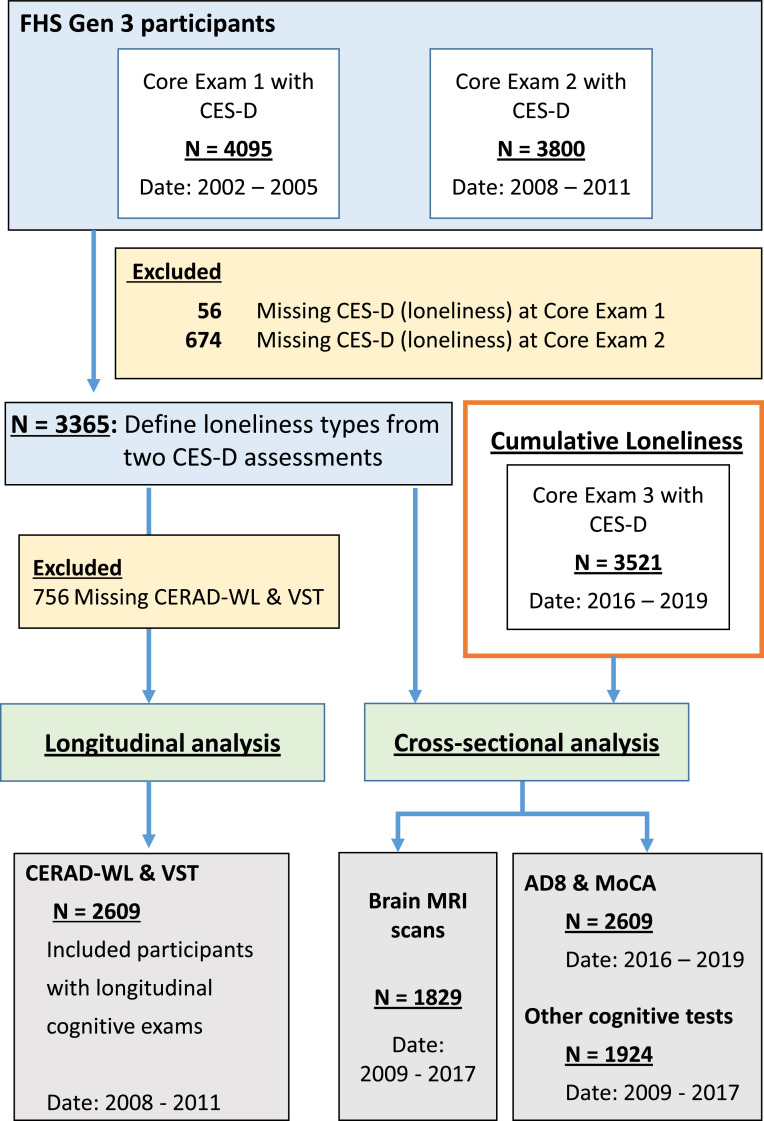
**Flow-chart of the study sample**. Age is reported in years with [95%CI] for each exam and subset. CERAD-WL and VST were longitudinally mearsured from 2008 to 2019. Other cognitive tests include Logical Memory, Visual Reproduction, Trails A and Trails B, and the Boston Naming Test. FHS Gen 3= Framingham Heart Study Generation 3. CES-D = Center for Epidemiologic Studies Depression Scale. CERAD-WL = CERAD Word List Memory Test. VST = Victoria Stroop Test. AD8 = Washington University Dementia Screening Test. MoCA = Montreal Cognitive Assessment.

FHS is a multi-generation, community-based, prospective cohort study in Framingham, Massachusetts. Participants from the third generation have been longitudinally examined in three core exams, on average every five years between 2002 to 2019. Details about this cohort have been previously described.^10^ This study selected participants who had loneliness assessments at two core exams (*n =* 3,365), core exam 1 (2002-2005) and core exam 2 (2008–2011), which we used to define loneliness types (described below). We excluded those who did not have two cognitive assessments, the CERAD Word List Memory Test (CERAD-WL) and the Victoria Stroop Test (VST), between 2008–2019 (*n =* 756). The final sample size included in this analysis consisted of 2,609 participants (Figure 1). The excluded participants showed no differences regarding age and sex but had lower education levels, compared to the analytical sample (*p*-value <0.001). Thus, we included education in all our analytical models. Participants completed the Washington University Dementia Screening Test (AD8 score) and the Montreal Cognitive Assessment (MoCA) at exam 3 (2016–2019) (*n =* 2,069), had detailed neuropsychological tests (NP) (*n =* 1,976), and magnetic resonance imaging (MRI) data (*n =* 1,833). For the subset analysis, Apolipoprotein *ApoE* genotype data was used (*n =* 2,488). A post-hoc power calculation showed a predicted power of 99% to detect small effect sizes. Written informed consent was obtained from all study participants. The study protocol was approved by the Boston University Institutional Review Board (reference numbers: H-40620, H-32132) and followed the Strengthening the Reporting of Observational Studies in Epidemiology (STROBE) reporting guideline.^11^

### Defining loneliness types

Participants completed the Center for Epidemiologic Studies Depression Scale (CES-D, 20 items) at core exams 1 and 2, including the self-reported item “I felt lonely during the past week”. The loneliness score was based on a 4-point Likert scale of number of days feeling lonely within the past week.^5^ We defined loneliness as feeling lonely at least 1–2 days within the past week and further differentiated between the following loneliness types:

• No loneliness: Not lonely at either exam 1 (2002–2005) or exam 2 (2008-2011);
• Incident loneliness: Lonely at exam 2 but not at exam 1;
• Transient loneliness: Lonely at exam 1 but not at exam 2;
• Persistent loneliness: Lonely at both exam 1 and exam 2.

### Cognitive assessments

Participants’ memory and executive function were assessed using the CERAD Word List Memory Test (CERAD-WL) and the Victoria Stroop Test (VST) at two core exams (core exam 2: 2008-2011; core examt 3: 2016-2019) (Figure 1). Higher scores on the CERAD indicate better memory, whereas lower scores on the VST indicate better executive function. Further, participants completed two test scores related to cognitive impairment, the AD8 score and the MoCA test (core exam 3: 2016–2019). Better memory performance is indicated by lower scores on the AD8 and higher scores on the MoCA. Between 2009 and 2017, along with the MRI scans, participants completed a NP test battery that provides a structured approach to the assessment of cognitive function. The entire NP test battery took 45-90 minutes and was administered by a trained research assistant. In this study, we used the following tests relating to different cognitive domains: logical and verbal memory (Wechsler Memory Scale Delayed Recall and Recognition) and visual memory (Visual Reproductions Delayed Recall and Recognition), visual-spatial and executive functioning (Trail Making Test (TMT) A and B), and language (30-item Boston Naming Test). A higher score on each subtest of the NP battery indicates better cognitive function (except for the TMT).

### Brain MRI measures

The FHS MRI protocol has been previously described.^12^ Briefly, participants were imaged by a 1.5T MRI (Siemens Medical, Erlangen, Germany) and used a 3-dimensional T1-weighted coronal spoiled gradient-recalled echo sequence. All images were transferred to and processed by the University of California Davis Medical Center without knowledge of clinical information. Segmentation and quantification of total cerebral cranial volume (TCV), total brain volume, frontal lobe (FBV), parietal lobe (PBV), occipital lobe (OBV), temporal lobe (TBV), hippocampus, and lateral ventricle volumes were performed using semi-automated procedures previously described.^12^, ^13^, ^14^ TCV was determined using a convolutional neural network method.^15^ Non-linear co-registration of images to the Desikan-Killiany-Tourville atlas^16^ enabled calculation of regional gray matter volumes.^17^^,^^18^ MRI volumes were corrected for head size by calculating the percentage of TCV. Each image set underwent rigorous quality control including assessments of the original acquisition and image processing quality.

### Statistical analyses

Analyses were performed using the statistical analysis software R statistical environment (2017).^19^ Next to assessing the assumptions of linearity, normality, and homoscedasticity by plotting residuals in Q-Q plots, we assessed normality using the Shapiro-Wilk's normality test and assessed homogeneity of variance using the Fligner-Killeen test. Mean + standard deviation (SD) and one way ANOVA were used for the variables with a normal distribution, and median (min, max) and Kurskal-Wallis tests were used for variables with a skewed distribution. The Chi-Square test was applied to analyze categorical data; Fisher's exact tests were used when one or more expected cell count in the cross-tabulation was less than 5. Cognitive test scores and MRI measures were rescaled to z-scores (Mean=0; SD=1). Next, we conducted longitudinal analyses between the different loneliness types and cognitive changes. Specifically, the longitudinal changes of the CERAD-WL and VST scores were calculated by **Δ** = scale [(scores at exam 3 - scores at exam 2)/(scores at exam 2)]). Using the *rlm* function from the *MASS* package,^20^ robust regression models were conducted with the three loneliness types as risk factors for changes in cognitive function (with no loneliness as the reference group). We applied robust regression models to deal with outliers and skewed data. To address issues of heterogeneity of variance, functions from the R *sandwich* package were used.^21^ Furthermore, we studied the cross-sectional associations between loneliness types and additional cognitive scores (i.e., AD8, MoCA, and NP test battery) as well as brain volumes.

Covariates were selected using literature review and directed acyclic graphs (Supplementary Figure S2). For all analyses, we used two models that controlled for different sets of covariates at baseline; Model 1 controlled for age, sex, education, and the time between the second loneliness assessment and the last cognitive test or MRI scan; Model 2 additionally controlled for *ApoE*4, current smoking, Body Mass Index (BMI), marital status, and employment. Listwise deletion was applied for the models with missing data. To estimate the effect modification of some common risk factors for dementia, we conducted stratified analyses for sex and depression status. Results were shown as beta estimates (*β*) with 95% confident intervals (95% CIs) along with *p*-values. Statistical significance was indicated by a *p*-value <0·05 (two-tailed tests). Effects sizes were estimated using the partial Cohen's *f^2^* (with 0.02, 0.15, and 0.35 indicating small, medium, and large effects, respectively).^22^

In a sensitivity analysis, we created a cumulative loneliness score over three exams to account for loneliness frequency across the three exams (Supplementary Table S1). Since the cumulative loneliness score for three exams ranged from 0 to 9 (4-point Likert scale from 0 to 3 for a single exam), a higher cumulative loneliness score represents higher frequency of loneliness over time. We tested the association between the cumulative loneliness scores with six MRI brain volumes, including FBV, PBV, OBV, TBV, hippocampus, and lateral ventricle volumes. Results were subsequently stratified for *ApoE*4 status.

### Role of the funding source

This study was supported by grants from the US National Institute on Aging. The sponsor institute did not play any role in design and conduct of the study; collection, management, analysis, and interpretation of the data; preparation, review, or approval of the manuscript; and decision to submit the manuscript for publication. QT and TFA had full access to the dataset and first authors QT and SA as well as last author WQ had final responsibility for the decision to submit the study for publication.

## Results

### Characteristics of loneliness types

The study sample had 2,609 participants with an average age of 46·3 ± 8·6 years, and 1,400 (54%) were female. Among them, 1,813 (69·5%) participants reported no loneliness, 219 (8·4%) experienced incident loneliness, 353 (13·5%) had transient loneliness, and 224 (8·6%) had persistent loneliness (Table 1). Participants who reported persistent loneliness were more likely to be female, be depressed, smoke, have a higher BMI, be single, unemployed, and have a shorter follow-up time to the MRI exams, compared with participants from other loneliness types (Table 1). Other variables including age, education, *ApoE* ε4 carrier status, CVD, diabetes, and times to cognitive tests did not differ significantly across the four loneliness types. Effect sizes for the following results ranged from small (*f^2^*<0.02 for MRI measures) to large (*f^2^*=0.04 for AD8 scores).

**Table 1:** Characteristics of the study sample by loneliness types.

Characteristics‡	Overall (N=2609)	Loneliness types				
		Not lonely (N=1813)	Incident (N=219)	Transient (N=353)	Persistent (N=224)	*P*-value
**Age (years)**						
Mean ± SD	46.3 ± 8.6	46.4 ± 8.4	45.9 ± 8.3	46.7 ± 9.2	45.5 ± 9.5	0.53
Median [Min, Max]	47 [24, 76]	47 [24, 76]	46 [25, 63]	48 [24, 71]	46 [24, 73]	0.49
**Female, n (%)**	1400 (54)	928 (51)	139 (64)	198 (56)	135 (60)	0.001
**Education, n (%)**						
High school or less	341 (13)	234 (13)	19 (9)	59 (17)	29 (13)	0.03
Some college	753 (29)	509 (28)	57 (26)	108 (31)	79 (35)	
College or higher	1512 (58)	1068 (59)	142 (65)	186 (53)	116 (52)	
***ApoE* ε4 carrier, n (%)**	555 (21)	397 (22)	56 (26)	60 (17)	42 (19)	0.16
**Depression, n (%)**†	214 (8)	34 (2)	62 (28)	23 (7)	95 (42)	<0.001
**CVD, n (%)**	56 (2)	34 (2)	5 (2)	9 (3)	8 (4)	0.54
**Stroke, n (%)**	8 (0)	5 (0)	0 (0)	0 (0)	3 (1)	0.15
**Current Diabetes, n (%)**	109 (4)	68 (4)	10 (5)	17 (5)	14 (6)	0.45
**Current Smoking, n (%)**	323 (12)	186 (10)	33 (15)	66 (19)	38 (17)	<0.001
**BMI (kg/m^2^)**						
Mean ± SD	27.8 ± 5.6	27.6 ± 5.5	27.6 ± 5.6	28.2 ± 5.6	29 ± 6.8	0.008
Median [Min, Max]	27 [16, 61]	27 [16, 50]	27 [16, 44]	28 [18, 54]	28 [18, 61]	0.03
**Marital status, n (%)**						
Single	367 (14)	186 (10)	39 (18)	67 (19)	75 (34)	<0.001
Married	1915 (73)	1452 (80)	135 (62)	224 (64)	104 (46)	
Other	324 (12)	174 (10)	45 (21)	61 (17)	44 (20)	
**Employment status, n (%)**						
Full time	1884 (72)	1345 (74)	148 (68)	251 (71)	140 (63)	<0.001
Part time	355 (14)	249 (14)	30 (14)	44 (13)	32 (14)	
Unemployed	88 (3)	51 (3)	6 (3)	14 (4)	17 (8)	
Other	280 (11)	166 (9)	35 (16)	44 (13)	35 (16)	
**Years to the last cognitive test#**						
Mean ± SD	7.9 ± 0.7	7.9 ± 0.7	7.9 ± 0.7	7.9 ± 0.7	8.0 ± 0.7	0.25
Median [Min, Max]	7.8 [5.5, 10.8]	7.8 [5.6, 10.8]	7.8 [5.7, 10.7]	7.8 [5.5, 10.6]	7.9 [6.5, 10.5]	0.09
**Years to the MRI exam**§						
Mean ± SD	4.5 ± 2.9	4.6 ± 2.9	4.9 ± 2.9	4.2 ± 2.8	4.2 ± 3.0	0.09
Median [Min, Max]	4.0 [-0.1, 9.4]	4.1 [0.0, 9.4]	6.1 [0.0, 9.1]	3.4 [-0.1, 9.3]	3.1 [0.0, 9.1]	0.03

The loneliness types were defined as: No loneliness = participants did not report loneliness at neither exam 1 nor exam 2; Transient loneliness = participants reported loneliness only at exam 1; Incident loneliness = participants reported loneliness only at exam 2; Persistent loneliness = participants reported loneliness at both exam 1 and exam 2.

CVD = Cardiovascular disease; BMI = Body Mass Index; MRI = Magnetic Resonance Imaging.

*P*-values were two-tailed. One-way ANOVA tests for continuous variables and Chi-square tests for categorical variables were applied to compare each variable among the four loneliness types. Fisher's exact test were applied when the cell counts were less than 5 for the two variables CVD and stroke. Kruskal-Wallis tests were applied for the variables with Median [Min, Max].

^♯^The time difference between the second loneliness exam (core exam 2) and the last cognitive tests (CREAD, VST, AD8 score, and MOCA) at core exam 3.

† Depression was defined as CES-D score ≥ 16, where the CES-D score was calculated based on 19 out of 20 items of the original CES-D questionnaire, after excluding the loneliness item.

‡ The following variables had missing data: Education *n =* 3 (0.1%), *ApoE* genotype *n =* 121 (4.6%), current diabetes *n =* 6 (0.2%), BMI *n =* 2 (0.1%), and employment *n =* 2 (0.1%).

§ The time difference between the dates of the second loneliness exam and the MRI scan.

### Association of loneliness types with longitudinal change in memory and executive function

CERAD and VST cognitive tests were conducted twice and the average time between assessments was 7·92 ± 0·68 years. At the first cognitive assessment, the participants were aged 46 (95%CI [30, 63]) years on average (Figure 1). Table 2 shows results from a robust linear regression model with the three loneliness types as predictors and longitudinal change in CERAD and VST scores as outcomes. Compared to no loneliness, persistent loneliness was associated with a decrease in the CERAD recall (*β* = −0·13, 95%CI [−0.25, 0.00], *P* = 0·04) and retention scores (*β* = −0·15, 95%CI [−0.27, −0.03], *P* = 0·01), but not with changes in the total score. Results were similar when controlling for additional covariates including *ApoE*4, current smoking, BMI, marital status, and employment status in Model 2 (Supplement Table S2). Moreover, persistent loneliness was associated with an increase in the VST interference score (*β* = 0·14, 95%CI [0.02, 0.26], *P*=0·02), compared to no loneliness. In both models, incident and transient loneliness were not associated with longitudinal changes in cognitive performance. When assessing the cross-sectional associations, we found that persistent loneliness was only associated with the VST dot time at baseline (*β* = 0·13, 95%CI [0.01, 0.25], *P*=0·03) (Model 1, Supplement Table S3).

**Table 2:** The associations between loneliness types and longitudinal changes of CERAD and VST tests.

Neurocognitive test scores	Incident loneliness		Transient loneliness		Persistent loneliness	
	*β* (95%CI)	*P* value	*β* (95%CI)	*P* value	*β* (95%CI)	*P* value
**CERAD-WL (*n =* 2606)**						
CERAD total score	−0.05 (−0.16, 0.07)	0.42	−0.02 (−0.13, 0.08)	0.67	−0.02 (−0.16, 0.11)	0.74
CERAD recall score	0.03 (−0.08, 0.14)	0.56	−0.03 (−0.12, 0.06)	0.53	−0.13 (−0.25, 0.00)	0.04
CERAD retention score	0.06 (−0.06, 0.18)	0.31	−0.06 (−0.16, 0.04)	0.22	−0.15 (−0.27, −0.03)	0.01
**VST (*n =* 2606)**						
Stroop dot time	0.02 (−0.08, 0.12)	0.69	−0.03 (−0.12, 0.06)	0.58	−0.08 (−0.18, 0.02)	0.12
Stroop color time	0.12 (0.00, 0.25)	0.05	0.06 (−0.04, 0.16)	0.25	0.09 (−0.04, 0.21)	0.18
Stroop interference score	0.08 (−0.04, 0.21)	0.17	0.03 (−0.07, 0.13)	0.53	0.14 (0.02, 0.26)	0.02

Robust regression models were used to study the relationship between loneliness types (reference group: no loneliness) as a risk factor and longitudinal changes (Δ = scale [(scores at exam 3 - scores at exam 2)/(scores at exam 2)]) of CERAD Word List Memory Test (CERAD-WL) and Victoria Stroop Test (VST). All models were adjusted for baseline age, sex, education, and the follow-up time (years).

### The associations of loneliness types with other cognitive measures

We investigated the cross-sectional relationship between loneliness types and the cognitive tests specific to cognitive impairment, including the AD8 and MoCA, since these cognitive measures were only assessed once (Figure 1). In the total sample, loneliness was associated with higher AD8 scores for all loneliness types (incident *β* = 0·17, 95%CI [0.06, 0.28], *P* = 0.002; transient *β* = 0·14, 95%CI [0.05, 0.23], *P* = 0.001; and persistent *β* = 0·47, 95%CI [0.32, 0.61], *P* < 0.001), compared to no loneliness (Table 3). However, only persistent loneliness was associated with lower MoCA scores compared to no loneliness, indicating worse cognitive performance (*β* = −0·19, 95%CI [−0.32, −0.06], *P* = 0·005, Table 3).

**Table 3:** The associations between loneliness types and the AD8 and MoCA scores stratified by sex.

Neurocognitive test scores	Incident loneliness		Transient loneliness		Persistent loneliness	
	β (95%CI)	*P* value	β (95%CI)	*P* value	β (95%CI)	*P* value
**All** (*n =* 2585)						
AD8 score	0.17 (0.06, 0.28)	0.002	0.14 (0.05, 0.23)	0.001	0.47 (0.32, 0.61)	<0.001
MoCA score	−0.06 (−0.19, 0.07)	0.35	−0.07 (−0.17, 0.03)	0.19	−0.19 (−0.32, −0.06)	0.005
**Female** (*n =* 1386)						
AD8 score	0.11 (−0.02, 0.25)	0.1	0.06 (−0.04, 0.17)	0.25	0.48 (0.27, 0.69)	<0.001
MoCA score	−0.14 (−0.29, 0.01)	0.07	−0.10 (−0.24, 0.04)	0.14	−0.27 (−0.45, −0.08)	0.005
**Male** (*n =* 1199)						
AD8 score	0.27 (0.09, 0.46)	0.004	0.26 (0.12, 0.41)	<0.001	0.45 (0.26, 0.64)	<0.001
MoCA score	0.06 (−0.17, 0.29)	0.62	−0.03 (−0.19, 0.12)	0.65	−0.09 (−0.28, 0.09)	0.32

Robust regression models were used to investigate the relationship between loneliness types as risk factors and the cognitive tests including the Washington University Dementia Screening Test (AD8, z-score) and Montreal Cognitive Assessment (MoCA) score (z-score) as outcomes. All models were adjusted for baseline age, sex, education, and time difference between exam 2 and exam 3.

Next, we applied robust regression models for each cognitive domain of the NP battery including logical memory, executive function, visual reproduction, and language as outcomes (Supplementary Table S4). Participants with persistent loneliness had the lowest cognitive scores in memory and executive function. After adjusting for covariates (Model 1), persistent loneliness was associated with worse performance on measures of logical memory (recognition) (*β* = −0·19, 95%CI [−0.36, −0.03], *P*=0·02), the reversed z-score of TMT A (*β* = −0·12, 95%CI [−0.24, −0.01], *P*=0·04) and visual reproduction (recognition) (*β* = −0·20, 95%CI [−0.35, −0.04], *P*=0·01), compared to no loneliness. Only the association of persistent loneliness with logical memory (recognition) remained significant after adjusting for *ApoE*4, current smoking, BMI, marital status, and employment (*β* = −0·22, 95%CI [−0.40, −0.04], *P*=0.01) in Model 2 (Supplementary Table S4). Unlike persistent loneliness, there were no associations between transient or incident loneliness and measures of cognitive function. Taken together, the persistent loneliness group showed stronger negative associations with cognitive measures, especially with logical memory, than the transient or incident loneliness group.

### Associations of loneliness types with brain structure

To study the relationship between loneliness types and brain volumes, we used a subsample (N=1,829) with available brain MRI data. After controlling for covariates, persistent loneliness was associated with smaller temporal lobe volume, compared to no loneliness (*β* = −0·18, 95%CI [−0.32, −0.04], *P*=0·01, Table 4). Incident and transient loneliness did not show any relationships with brain volumes.

**Table 4:** The associations between loneliness types and brain volumes stratified by sex.

MRI Brain volumes	Incident loneliness		Transient loneliness		Persistent loneliness	
	β (95%CI)	*P* value	β (95%CI)	*P* value	β (95%CI)	*P* value
All subjects (*n =* 1829)						
Frontal Lobe	−0.07 (−0.23, 0.08)	0.36	−0.10 (−0.23, 0.03)	0.12	−0.08 (−0.21, 0.06)	0.27
Temporal Lobe	−0.04 (−0.19, 0.12)	0.65	−0.01 (−0.13, 0.10)	0.81	−0.18 (−0.32, −0.04)	0.01
Hippocampus Volume	0.10 (−0.06, 0.27)	0.23	−0.01 (−0.15, 0.13)	0.88	−0.12 (−0.27, 0.03)	0.12
Lateral Ventricle Volume	0.07 (−0.04, 0.18)	0.22	0.07 (−0.02, 0.17)	0.13	0.06 (−0.05, 0.16)	0.29
Female (*n =* 979)						
Frontal Lobe	−0.18 (−0.38, 0.02)	0.08	−0.19 (−0.35, −0.02)	0.02	−0.19 (−0.38, −0.01)	0.04
Temporal Lobe	−0.08 (−0.27, 0.12)	0.45	0.02 (−0.14, 0.17)	0.84	−0.20 (−0.37, −0.03)	0.02
Hippocampus Volume	−0.02 (−0.21, 0.17)	0.84	−0.02 (−0.20, 0.17)	0.85	−0.23 (−0.40, −0.06)	0.007
Lateral Ventricle Volume	0.15 (0.02, 0.28)	0.03	0.12 (0.00, 0.23)	0.06	0.15 (0.02, 0.28)	0.03
Male (*n =* 850)						
Frontal Lobe	0.06 (−0.18, 0.29)	0.63	−0.01 (−0.21, 0.18)	0.88	0.09 (−0.12, 0.29)	0.40
Temporal Lobe	0.05 (−0.21, 0.30)	0.71	−0.06 (−0.24, 0.13)	0.53	−0.15 (−0.39, 0.10)	0.24
Hippocampus Volume	0.30 (0.03, 0.58)	0.03	0.00 (−0.22, 0.22)	1.00	0.08 (−0.21, 0.37)	0.58
Lateral Ventricle Volume	−0.05 (−0.25, 0.16)	0.66	0.03 (−0.11, 0.17)	0.7	−0.09 (−0.25, 0.07)	0.26

Robust regression models were used to study the relationship between loneliness types as risk factors and MRI brain volumes (z-scores) as outcomes. All models were adjusted for baseline age, sex, education, and the follow-up time from the loneliness assessments at exam 2 and the brain MRI scan.

**Figure 2:**
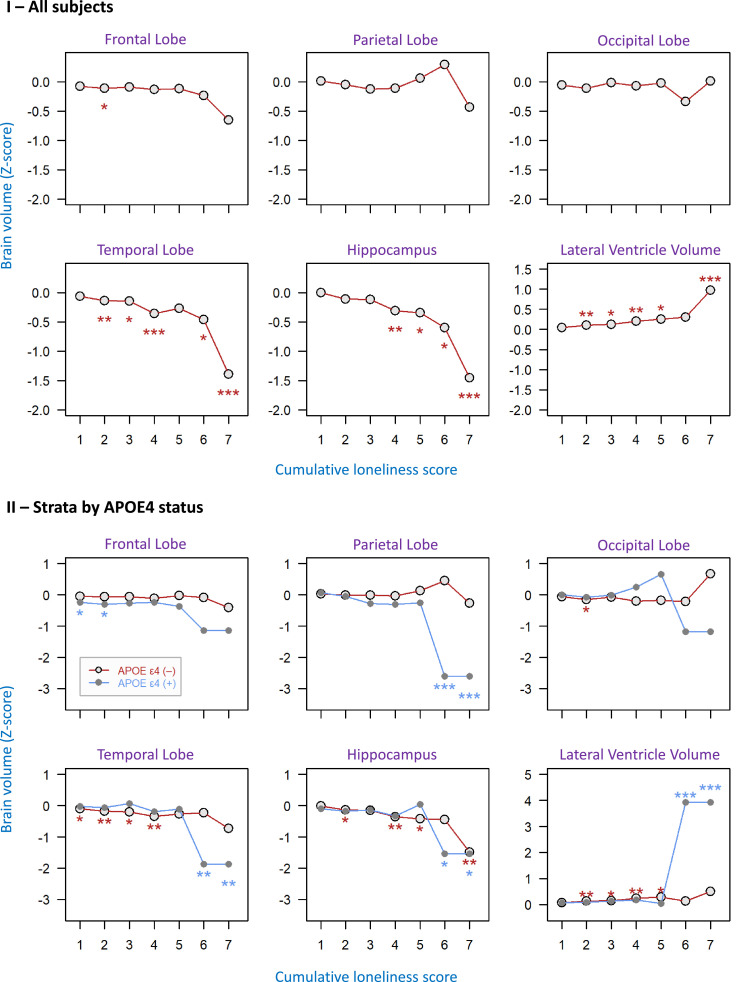
**The dose-response relationship between cumulative loneliness and brain volumes**. The loneliness scores across three exams were added to get a cumulative score for loneliness for each participant. A higher score indicates higher chronicity of loneliness over three exams. Robust linear regression models were used to study the relationship between cumulative loneliness scores and brain volumes as outcomes. The outcomes were the z-scores of MRI brain volumes adjusted for head size (y-axis); the predictors were the longitudinal cumulative loneliness scores (CLS) across three exams. Specifically, the labels (1 to 7) of the x-axis were doses of loneliness frequency, which were defined as the sum score of the 4-point Likert scale (0-3) across three exams. Panel (I) shows all subjects, whereas in Panel (II), the sample was stratified by *ApoE4* carrier status. All models were adjusted for baseline age, sex, education, and the time difference between the dates of the last loneliness exam (exam 3) and the MRI scans. Statistical significance was indicated as *p < 0.05, **p < 0.01, ***p < 0.001

Having three exams that assess loneliness, we used a sensitivity analysis to test the association between the cumulative loneliness score over three exams with six brain volumes (Figure 1). We tested the dose-response effects of loneliness scores on brain volumes using robust linear regression analyses (Figure 2). An increase in the cumulative loneliness score was related with smaller temporal lobe and hippocampus volumes and larger lateral ventricle volume in a dose-response pattern (β values) when controlled for age, sex, education, and time between exams. These relationships remained after adding the covariates of *ApoE4*, current smoking, BMI, marital status, and employment in Model 2 (data not shown). A particularly steep change in regional brain volumes was found between the loneliness values of 6 and 7 (Panel I of Figure 2). There was no significant relationship between cumulative loneliness and occipital lobe volume (Panel I of Figure 2). Consistent with the relationship between loneliness, temporal lobe, and hippocampus, incrementally higher cumulative loneliness was associated with incrementally higher AD8 scores and a trend towards lower MoCA scores (Supplementary Figure S1).

### Results stratified by sex, ApoE genotype, and depression status

The proportion of women was higher in the loneliness groups, specifically among incident (N = 139, 64%) and persistent loneliness (N = 135, 60%) (Table 1). Among women, while there were no significant changes in cognitive function (CERAD and VST) over time when controlling for all covariates including *ApoE4*, current smoking, BMI, marital status, and employment (Model 2; Supplement Table S5), persistent loneliness was associated with lower MoCA scores (Table 3). Similarly, persistent loneliness, but not transient and incident loneliness, was associated with AD8 scores (Supplement Table S6). On the contrary, among men, persistent loneliness was associated with longitudinal decline in the CERAD recall score (*β* = −0·30, 95%CI [−0.56, −0.04], *P* = 0·02) and CERAD retention score (*β* = −0·30, 95%CI [−0.55, −0.06], *P* = 0·01); and longitudinal increase in VST Stroop color time (*β* = 0·33, 95%CI [0.09, 0.58], *P* = 0·01) and VST interference scores (*β* = 0·32, 95%CI [0.09, 0.55], *P* = 0·01) (Supplement Table S5), while all three loneliness types were associated with the AD8 but not with the MoCA (Supplement Table S6).

Sex differences were also found in the relationship of loneliness types with brain volumes. Among women, persistent loneliness was associated with smaller temporal lobe volume (*β = −*0·20, 95%CI [−0.37, −0.03], *P* = 0.02), smaller hippocampus volume (*β =* −0·23 95%CI [−0.40, −0.06], *P*=0·007), and smaller frontal lobe volume (*β =* −0·19 95%CI [−0.38, −0.01], *P*=0·04, Table 4). Among women, all three loneliness types were associated with larger ventricle volumes. In contrast, among men, loneliness types were not found to be associated with brain volumes, except for the association between incident loneliness and larger hippocampus volume (*β* = 0·30, 95%CI [0.03, 0.58], *P* = 0·03, Table 4). This relationship among men remained when controlling for all covariates in Model 2 (data not shown).

As *ApoE*4 is a well-known genetic risk factor for cognitive decline and AD risk, we hypothesized that the relationship between persistent loneliness and brain atrophy would be even stronger in the presence of *ApoE*4. Indeed, Figure 2 (Panel II) shows stronger dose-response relationships between a higher cumulative loneliness score and smaller temporal lobe and hippocampus volumes as well as larger lateral ventricle volume in *ApoE*4 carriers, compared to *ApoE*4 non-carriers, when controlled for covariates. Particularly, with higher cumulative loneliness, a strong decline in parietal lobe volume was found only among *ApoE*4 carriers.

Lastly, we investigated whether loneliness types were associated with cognition and brain volumes independent of depression by stratifying the results. The adjusted CES-D score was calculated based on 19 out of 20 items from the original CES-D questionnaire after excluding the loneliness item (with a cut-off of ≥16 indicating depressive symptoms). Among participants who were not depressed, persistent loneliness was associated with longitudinal changes in memory and executive function, while both transient (*β* = 0·13, 95%CI [0.04, 0.22], *P* = 0·004) and persistent (*β* = 0·27, 95%CI [0.12, 0.42], *P* <0·001) loneliness were associated with the AD8 (Supplement Table S6). Among participants with depressive symptoms, there were no significant associations between loneliness groups and CERAD or VST scores (Supplement Table S5), however, persistent loneliness was associated with AD8 scores (*β* = 0·59, 95%CI [0.10, 1.09], *P* = 0·02). The MoCA was not significantly related to loneliness among either stratified depression group (Supplement Table S6).

## Discussion

In this study, we investigated associations between different loneliness types, cognitive function, and brain volumes in midlife using data from the FHS’ third generation. Our results suggest that the persistence of loneliness exacerbates cognitive decline and may pose a threat to brain health in the aging process, especially in the presence of *ApoE*4. A potential future increase of loneliness as a consequence of population aging and social isolation does not only make loneliness one of the most important public health concerns but may also have detrimental effects on people's future brain health and wellbeing.

First, we found that persistent loneliness in midlife was more strongly associated with cognitive decline—specifically the domains of logical memory and executive functioning—compared to transient or incident loneliness. This finding is in line with our hypothesis and results from a recent study conducted in China.^6^ Consistently, using data from the second generation of the FHS, we previously found that persistent loneliness was a risk factor for AD dementia, whereas transient loneliness was a protective factor.^5^ The current study cohort (FHS Gen 3) is younger, with an average age of 46 years. Although participants were generally too young to develop AD, we observed associations between loneliness and cognitive decline in domains consistent with preclinical AD. Based on these results, we posit that people who recover from loneliness (i.e., transient loneliness) are somewhat resilient to the adverse effect of loneliness on cognition.

Second, our study found that persistent loneliness was associated with smaller temporal lobe volume. Additionally, a higher cumulative loneliness score was associated with smaller brain volumes in the hippocampus and with enlarged lateral ventricle in a “dose-dependent” pattern, especially in ApoE4 carriers. Our findings are consistent with previously published studies,^23^ showing that loneliness was associated with areas in the prefrontal, hippocampal, and temporal regions. Using functional MRI (fMRI), loneliness was shown to be associated with areas in the default mode network of the brain, regulating mood and cognition (i.e., including temporal areas, the medial prefrontal cortex, the anterior cingulate cortex, the temporoparietal junction, and posteromedial parietal cortex).^9^^,^^24^ These areas largely overlap with areas belonging to the “social brain”^24^ and are known to be associated with reminiscence, imagination, mentalizing as well as age-related cognitive decline and AD pathology. Interestingly, our study showed that long-term cumulative loneliness was associated with parietal lobe atrophy only in the presence of *ApoE*4, a strong genetic risk factor for AD. Several studies have shown that parietal lobe atrophy plays a significant role in the early stage of AD development.^25^

Consistent with women being at higher risk for feeling lonely and developing AD, our study found that relationships between persistent loneliness, cognitive decline, and brain structures, especially the hippocampus and temporal lobe, were stronger among women. We also found that loneliness, especially among women, was associated with larger ventricle volume. Larger ventricle volume was found to be associated with slower response inhibition, an essential subcomponent of executive function closely related to AD progression.^26^ Prior studies have reported gender differences in neural correlates of loneliness. Our results are in line with a recent study including over 10,000 participants,^27^ showing that sensory network brain volumes diverged in lonely versus non-lonely women, but not in men. Yet, another study found a stronger relationship of loneliness with white matter volume among men.^28^ Taken together, these results motivate future neuroimaging studies to investigate sex differences in the relationship between loneliness and brain structure and function, specifically regarding hippocampal regions.

Overall, the findings suggest that persistent loneliness—resulting from a combination of genetic background, social environment, and coping skills—may be related to reduced brain health. Although the loneliness assessments across the exams were years apart, results may reflect personality differences when facing life stressors. For instance, persistent loneliness was associated with current smoking and a higher BMI, indicating lower self-care behavior. Still, there are some limitations to this study. First, this study is merely observational, cannot eliminate the issue of reverse causality, and does not allow causal inference.^29^ In fact, previous studies suggest that loneliness and brain health are likely to be related via bidirectional pathways.^30^^,^^31^ Further, we only had longitudinal assessments of two cognitive measures (CERAD and VST), whereas other cognitive tests only had one assessment, limiting the longitudinal analysis of loneliness with each cognitive domain. Second, the third FHS generation does not have assessments of social isolation. However, using data from the second FHS generation, which includes measures of social isolation, we found that living alone did not influence the relationship between persistent loneliness and AD risk.^5^ Third, although a single item has been argued to be a trustworthy assessment for measuring loneliness,^32^ future studies will benefit from implementing more comprehensive scales that assess different dimensions of loneliness. Fourth, the AD8 test also includes items related to emotional well-being,^33^ which could explain why loneliness was more associated with the AD8 compared to other cognitive tests in our study. For example, less interest in hobbies that involve social activities in the AD8 test may also be a symptom or consequence of loneliness,^34^ as people may feel excluded or afraid of being rejected. Future studies will need to be cautious of the assessment of different conditions, such as depression, loneliness, and AD, as these may have common underlying constructs.^35^ Moreover, we did not consider the time-varying nature of confounding factors such as BMI to identify the possible interaction with loneliness over time. Finally, all participants were white US-Americans, limiting the generalizability of our findings to other populations.

Nevertheless, our study, among others, sets a foundation for the early diagnosis and treatment of chronic loneliness, and ultimately, the early prevention and intervention of cognitive decline and AD. Future studies will benefit from investigating the relationship between persistent loneliness and amyloid pathology^36^ to fully understand biological mechanisms between chronic loneliness and brain health. Our study also raises questions for future research about whether and which socio-environmental factors can help people become resilient to persistent states or negative effects of loneliness on brain health. One way to investigate this question would be to implement additional methods in longitudinal cohort studies, such as qualitative interviews about coping techniques^4^ or remote activity monitoring using innovative technologies.^37^

## Contributors

QT and SA contributed equally to the manuscript. WQQ was the principal investigator for the study. QT, SA, and WQQ were responsible for the study design, data interpretation, and manuscript writing. QT, SA, TFA, II, and WQQ worked on the literature search and data analyses. TFA, CD, SD, and RA were involved in the FHS data collection process. CD, MA, JM, RK, SD, AR, XZ, KL, DS, LF, DG, and RA contributed to data interpretation and manuscript writing.

## Data sharing statement

The FHS has a long and strong tradition of open access data sharing to outside investigators with approved research proposals and Institutional Review Board protocols. Please visit the FHS website https://framinghamheartstudy.org/ for the guideline to obtain data. The FHS data for this study are publicly available.

## Declaration of interests

All authors claim no financial relationships with commercial interests.

## Appendix Supplementary materials

Image, application 1

Image, application 2

## References

[1] Peplau LA, Perlman D. (1982).

[2] Holt-Lunstad J, Smith TB, Baker M, Harris T, Stephenson D. (2015). Loneliness and social isolation as risk factors for mortality: a meta-analytic review. Perspect Psychol Sci.

[3] Valtorta NK, Kanaan M, Gilbody S, Ronzi S, Hanratty B. (2016). Loneliness and social isolation as risk factors for coronary heart disease and stroke: systematic review and meta-analysis of longitudinal observational studies. Heart.

[4] Deckx L, van den Akker M, Buntinx F, van Driel M. (2018). A systematic literature review on the association between loneliness and coping strategies. Psychol, Health Med.

[5] Akhter-Khan SC, Tao Q, Ang TFA (2021). Associations of loneliness with risk of Alzheimer's disease dementia in the Framingham Heart Study. Alzheimer's Dementia.

[6] Zhong BL, Chen SL, Conwell Y. (2016). Effects of transient versus chronic loneliness on cognitive function in older adults: findings from the chinese longitudinal healthy longevity survey. Am J Geriatr Psychiatry.

[7] Lee S, Charles ST, Almeida DM. (2021). Change is good for the brain: activity diversity and cognitive functioning across adulthood. Gutchess A, editor. J Gerontol Ser B.

[8] Li BJ, Friston K, Mody M, Wang HN, Lu HB, Hu DW. (2018). A brain network model for depression: from symptom understanding to disease intervention. CNS Neurosci Ther.

[9] Spreng RN, Dimas E, Mwilambwe-Tshilobo L (2020). The default network of the human brain is associated with perceived social isolation. Nat Commun.

[10] Splansky GL, Corey D, Yang Q (2007). The third generation cohort of the national heart, lung, and blood institute's framingham heart study: design, recruitment, and initial examination. Am J Epidemiol.

[11] von Elm E, Altman DG, Egger M (2007). The strengthening the reporting of observational studies in epidemiology (STROBE) statement: guidelines for reporting observational studies. Lancet.

[12] DeCarli C, Massaro J, Harvey D (2005). Measures of brain morphology and infarction in the framingham heart study: establishing what is normal. Neurobiol Aging.

[13] DeCarli C, Miller BL, Swan GE (1999). Predictors of brain morphology for the men of the NHLBI twin study. Stroke.

[14] Carmichael O, Mungas D, Beckett L (2012). MRI predictors of cognitive change in a diverse and carefully characterized elderly population. Neurobiol Aging.

[15] Fletcher E, DeCarli C, Fan AP, Knaack A. (2021). Convolutional neural net learning can achieve production-level brain segmentation in structural magnetic resonance imaging. Front Neurosci.

[16] Desikan RS, Ségonne F, Fischl B (2006). An automated labeling system for subdividing the human cerebral cortex on MRI scans into gyral based regions of interest. Neuroimage.

[17] Aljabar P, Heckemann RA, Hammers A, Hajnal JV, Rueckert D. (2009). Multi-atlas based segmentation of brain images: Atlas selection and its effect on accuracy. Neuroimage.

[18] Rueckert D, Aljabar P, Heckemann RA, Hajnal JV, Hammers A., Larsen R, Nielsen M, Sporring J (2006). Medical Image Computing and Computer-Assisted Intervention – MICCAI 2006.

[19] R Core Team (2020). https://www.R-project.org/.

[20] Venables WN, Ripley BD. (2002).

[21] Zeileis A, Köll S, Graham N. (2020). Various versatile variances: an object-oriented implementation of clustered covariances in *R*. J Stat Soft.

[22] Cinelli C, Hazlett C. (2020). Making sense of sensitivity: extending omitted variable bias. J R Stat Soc B.

[23] Lam JA, Murray ER, Yu KE (2021). Neurobiology of loneliness: a systematic review. Neuropsychopharmacol.

[24] Mars RB, Neubert FX, Noonan MP, Sallet J, Toni I, Rushworth MFS. (2012). On the relationship between the “default mode network” and the “social brain. Front Hum Neurosci.

[25] Jacobs HIL, Van Boxtel MPJ, Jolles J, Verhey FRJ, Uylings HBM. (2012). Parietal cortex matters in Alzheimer's disease: an overview of structural, functional and metabolic findings. Neurosci Biobehav Rev.

[26] Lundervold AJ, Vik A, Lundervold A. (2019). Lateral ventricle volume trajectories predict response inhibition in older age—a longitudinal brain imaging and machine learning approach. Jäncke L, editor. PLoS One.

[27] Kiesow H, Dunbar RIM, Kable JW (2020). 10,000 social brains: sex differentiation in human brain anatomy. Sci Adv.

[28] van der Velpen IF, Melis RJF, Perry M, Vernooij-Dassen MJF, Ikram MA, Vernooij MW. (2021). Social health is associated with structural brain changes in older adults: the rotterdam study. Biol Psychiatry.

[29] Tennant PWG, Arnold KF, Ellison GTH, Gilthorpe MS. (2021). Analyses of ‘change scores’ do not estimate causal effects in observational data. Int J Epidemiol.

[30] Akhter-Khan SC, Au R. (2020). Why loneliness interventions are unsuccessful: a call for precision health. Adv Geriatr Med Res.

[31] Yin J, Lassale C, Steptoe A, Cadar D. (2019). Exploring the bidirectional associations between loneliness and cognitive functioning over 10 years: the English longitudinal study of ageing. Int J Epidemiol.

[32] Mund M, Maes M, Drewke PM, Gutzeit A, Jaki I, Qualter P. (2022). Would the real loneliness please stand up? The validity of loneliness scores and the reliability of single-item scores. Assessment.

[33] Wei YC, Huang LY, Lin C, Shyu YC, Chen CK. (2021). Taiwanese depression questionnaire and AD8 questionnaire for screening late-life depression in communities. NDT.

[34] Queen TL, Stawski RS, Ryan LH, Smith J. (2014). Loneliness in a day: Activity engagement, time alone, and experienced emotions. Psychol Aging.

[35] Liu T, Lu S, Leung DKY (2020). Adapting the UCLA 3-item loneliness scale for community-based depressive symptoms screening interview among older Chinese: a cross-sectional study. BMJ Open.

[36] d'Oleire Uquillas F, Jacobs HIL, Biddle KD (2018). Regional tau pathology and loneliness in cognitively normal older adults. Transl Psychiatry.

[37] Austin J, Dodge HH, Riley T, Jacobs PG, Thielke S, Kaye J. (2016). A smart-home system to unobtrusively and continuously assess loneliness in older adults. IEEE J Transl Eng Health Med.

