# Supplementary material for: Different loneliness types, cognitive function, and brain structure in midlife: Findings from the Framingham Heart Study
Source: eClinicalMedicine. 2022 Sep 6;53:101643. doi: 10.1016/j.eclinm.2022.101643 (PMC9465265; doi:10.1016/j.eclinm.2022.101643)
Supplement: Supplementary file 1 [file mmc1.docx]

**Captions for supplementary material**

**Supplement Table S1. The sample sizes at each value of the cumulative loneliness score**

A cumulative loneliness score was created based on the loneliness score in three core exams. The score for sample *i* is defined as $S_{i}= \sum_{j=1}^{m} x_{ij}$, where m is the number of loneliness exams (m = 3) and *x*_ij_ is the loneliness score j for sample i. The maximum cumulative loneliness score over three exams is 9, since the loneliness score item response was indicated on a 4-point Likert Scale (0-3) at each exam.

**Supplement Table S2. The associations between loneliness and longitudinal CERAD and VST score changes**

Robust regression models were used to study the relationship between loneliness types (versus no loneliness) as risk factors and longitudinal changes (**Δ** = scale [(scores at exam 3 - scores at exam 2)/(scores at exam 2)]) of CERAD-WL and VST as outcomes. All models were adjusted for baseline age, sex, education, *ApoE*4, current smoking, BMI, marital status, employment status, and the follow-up times (years) between exam 2 and exam 3.

**Supplement Table S3. The associations between loneliness types and the baseline CERAD and VST scores**

Robust regression models were used to study the relationship between loneliness types (versus no loneliness) as risk factors and the baseline CERAD-WL and VST as outcomes (the original scores were transformed into z-scores). **Model 1** adjusted for baseline age, sex, and education. **Model 2** adjusted for baseline age, sex, education, *ApoE*4, current smoking, BMI, marital status, and employment status.

**Supplement Table S4. The association between loneliness and cognitive function (NP battery tests)**

Robust regression models were used to study the association between loneliness and neuropsychological (NP) tests including Logical Memory (LM), Trail Making Test (TMT), Visual Reproduction (VR), and Boston Naming Test. The outcomes were z-scores of NP tests; the predictors were the three loneliness types versus the no loneliness group. † A higher score on each subtest of the NP battery indicated better cognitive performance, except for the original TMT scores. Therefore, we revised the two TMT scores (TMT A and TMT B) by multiplying them by “-1”. Model 1 was adjusted for baseline age, sex, education, and the time between the second loneliness evaluation and the date of NP tests. Model 2 was adjusted for baseline age, sex, education, *ApoE*4, current smoking, BMI, marital status, employment status, and the time difference between the dates of the second loneliness assessment at exam 2 and the NP tests. Results are shown as regression coefficients and standard errors: β (SE). *p value < 0.05.

**Supplement Table S5. The associations between loneliness and longitudinal changes in cognitive function (Δ) stratified by sex and depression status**

Participants were divided into women versus men; no depression versus depressive symptoms for the stratified analyses. After the loneliness item was excluded from the CES-D, no depression versus depression was defined by an adjusted CES-D score < 16 versus ≥ 16. Robust regression models were used to study the relationship between loneliness types and longitudinal changes of NP tests including the CERAD-WL and VST. The outcomes were the longitudinal cognitive test changes (Δ = scale [(scores at exam 3 - scores at exam 2)/(scores at exam 2)]) of CERAD-WL and VST; the predictors were the three loneliness types versus the no loneliness group. All models were adjusted for baseline age, sex, education, *ApoE*4, current smoking, BMI, marital status, employment status, and time difference between the dates of the cognitive tests at exam 2 and exam 3.

**Supplement Table S6. The associations between loneliness and AD8 and MoCA scores stratified by sex and depression status**

Participants were divided into women versus men; no depression versus depressive symptoms for the stratified analyses. After the loneliness item was excluded from the CES-D, no depression versus depression was defined by an adjusted CES-D score < 16 versus ≥ 16. Robust regression models were used to study the relationship between loneliness types as risk factors and the follow-up tests including the AD8 (z-score) and MoCA score (z-scores) as outcomes. The outcomes were NP tests at exam 3; the predictors were the three loneliness types versus the no loneliness group. All models were adjusted for baseline age, sex, education, *ApoE*4, current smoking, BMI, marital status, employment status, and time difference between the dates of the cognitive tesst at exam 2 and exam 3.

**Supplement Figure S1. The dose-response relationship between cumulative loneliness and AD8 Score and MoCA score**

The loneliness scores across three exams were added to get a cumulative score for loneliness for each participant. A higher score indicates higher chronicity of loneliness over three exams. A linear regression model was used to study the relationship between cumulative loneliness scores and brain volumes as outcomes. The outcomes were the z-scores of AD8 Score and MoCA score respectively. the predictors were the longitudinal cumulative loneliness scores (CLS) across three exams with seven cut-off values. Specifically, the labels (1 to 7) of the x-axis were doses of loneliness frequency, which were defined as the sum score of the 4-point Likert scale (0-3) across three exams. Panel (I) show all subjects, whereas in Panel (II) the sample was stratified by *ApoE* ε4 carrier status. All models were adjusted for baseline age, sex, education, and the time difference between the dates of the last loneliness exam (exam 3) and the MRI scans. Statistical significance was indicated as *p < 0.05, **p < 0.01, ***p < 0.001.

**Supplement Figure S2. Directed Acyclic Graph**

Covariates were selected according to the directed acyclic graph (DAG), created with DAGitty tools (<http://www.dagitty.net/>). The minimal sufficient adjustment set used in all models includes age, sex, education, and time difference between the exams.
