# Supplementary material for: Different loneliness types, cognitive function, and brain structure in midlife: Findings from the Framingham Heart Study
Source: eClinicalMedicine. 2022 Sep 6;53:101643. doi: 10.1016/j.eclinm.2022.101643 (PMC9465265; doi:10.1016/j.eclinm.2022.101643)

**Supplement figures and tables**

Table of Contents

[Supplement Table S1. The sample sizes at each value of the cumulative loneliness score 2](#_Toc110787433)

[Supplement Table S2. The associations between loneliness and longitudinal CERAD and VST score changes 3](#_Toc110787434)

[Supplement Table S3. The associations between loneliness types and the baseline CERAD and VST scores 4](#_Toc110787435)

[Supplement Table S4. The association between loneliness and cognitive function (NP battery tests) 5](#_Toc110787436)

[Supplement Table S5. The associations between loneliness and longitudinal changes in cognitive function (Δ) stratified by sex and depression status. 6](#_Toc110787437)

[Supplement Table S6. The associations between loneliness and AD8 and MoCA scores stratified by sex and depression status 7](#_Toc110787438)

[Supplementary Figure S1. The dose-response relationship between cumulative loneliness and AD8 Score and MoCA score. 8](#_Toc110787439)

[Supplementary Figure S2. Directed Acyclic Graph. 9](#_Toc110787440)

# **Supplement Table S1. The sample sizes at each value of the cumulative loneliness score**

| *The composite loneliness score* | < value  n (%) | ≥ value  n (%) | Total  (N) |
| --- | --- | --- | --- |
| *0* | 0 (0.0%) | 2609 (100%) | 2609 |
| *1* | 1657 (63.5%) | 952 (36.5%) | 2609 |
| *2* | 2119 (81.2%) | 490 (18.8%) | 2609 |
| *3* | 2377 (91.1%) | 232 (8.9%) | 2609 |
| *4* | 2495 (95.6%) | 114 (4.4%) | 2609 |
| *5* | 2557 (98.0%) | 52 (2.0%) | 2609 |
| *6* | 2588 (99.2%) | 21 (0.8%) | 2609 |
| *7* | 2599 (99.6%) | 10 (0.4%) | 2609 |
| *8* | 2605 (99.8%) | 4 (0.2%) | 2609 |
| *9* | 2609 (100%) | 0 (0.0%) | 2609 |

# **Supplement Table S2. The associations between loneliness and longitudinal CERAD and VST score changes**

| **Cognitive test scores** | **Incident loneliness** | |  | **Transient loneliness** | |  | **Persistent loneliness** | |
| --- | --- | --- | --- | --- | --- | --- | --- | --- |
|  | *β* (95%CI) | P value |  | *β* (95%CI) | P value |  | *β* (95%CI) | P value |
| **CERAD-WL (n = 2480)** |  |  |  |  |  |  |  |  |
| CERAD total score | -0.02 (-0.14, 0.11) | 0.80 |  | -0.02 (-0.13, 0.10) | 0.79 |  | 0.00 (-0.14, 0.14) | 0.96 |
| CERAD recall score | 0.01 (-0.09, 0.12) | 0.81 |  | -0.04 (-0.13, 0.05) | 0.41 |  | -0.14 (-0.26, -0.01) | 0.03 |
| CERAD retention score | 0.04 (-0.08, 0.16) | 0.52 |  | -0.06 (-0.16, 0.04) | 0.22 |  | -0.15 (-0.27, -0.02) | 0.02 |
| **VST (n = 2480)** |  |  |  |  |  |  |  |  |
| Stroop dot time | 0.01 (-0.09, 0.12) | 0.78 |  | -0.01 (-0.11, 0.08) | 0.78 |  | -0.08 (-0.19, 0.03) | 0.14 |
| Stroop color time | 0.13 ( 0.01, 0.26) | 0.04 |  | 0.07 (-0.04, 0.17) | 0.22 |  | 0.12 (-0.02, 0.25) | 0.09 |
| Stroop interference score | 0.09 (-0.04, 0.21) | 0.16 |  | 0.03 (-0.08, 0.13) | 0.63 |  | 0.16 ( 0.03, 0.29) | 0.01 |

# **Supplement Table S3. The associations between loneliness types and the baseline CERAD and VST scores**

| **Neurocognitive test scores** | **Incident loneliness** | |  | **Transient loneliness** | |  | **Persistent loneliness** | |
| --- | --- | --- | --- | --- | --- | --- | --- | --- |
|  | *β* (95%CI) | P value |  | *β* (95%CI) | P value |  | *β* (95%CI) | P value |
| **Model 1 (n = 2606)** |  |  |  |  |  |  |  |  |
| **CERAD-WL** |  |  |  |  |  |  |  |  |
| CERAD total score | 0.03 (-0.11, 0.17) | 0.67 |  | 0.02 (-0.09, 0.13) | 0.76 |  | -0.08 (-0.21, 0.05) | 0.23 |
| CERAD recall score | -0.04 (-0.19, 0.10) | 0.54 |  | 0.06 (-0.06, 0.18) | 0.33 |  | 0.04 (-0.09, 0.16) | 0.59 |
| CERAD retention score | -0.06 (-0.18, 0.05) | 0.28 |  | 0.07 (-0.03, 0.18) | 0.19 |  | 0.05 (-0.06, 0.17) | 0.34 |
| **VST** |  |  |  |  |  |  |  |  |
| Stroop dot time | -0.02 (-0.14, 0.09) | 0.70 |  | 0.08 (-0.02, 0.17) | 0.13 |  | 0.13 ( 0.01, 0.25) | 0.03 |
| Stroop color time | -0.02 (-0.13, 0.08) | 0.66 |  | 0.00 (-0.09, 0.09) | 0.98 |  | 0.11 (-0.01, 0.23) | 0.07 |
| Stroop interference score | -0.04 (-0.15, 0.08) | 0.52 |  | -0.07 (-0.17, 0.03) | 0.16 |  | 0.01 (-0.10, 0.13) | 0.82 |
| **Model 2 (n = 2480)** |  |  |  |  |  |  |  |  |
| **CERAD-WL** |  |  |  |  |  |  |  |  |
| CERAD total score | 0.05 (-0.09, 0.20) | 0.48 |  | 0.01 (-0.10, 0.13) | 0.80 |  | -0.08 (-0.22, 0.06) | 0.25 |
| CERAD recall score | -0.01 (-0.16, 0.14) | 0.94 |  | 0.07 (-0.06, 0.20) | 0.28 |  | 0.07 (-0.07, 0.20) | 0.33 |
| CERAD retention score | -0.03 (-0.16, 0.09) | 0.58 |  | 0.08 (-0.03, 0.19) | 0.16 |  | 0.08 (-0.04, 0.20) | 0.20 |
| **VST** |  |  |  |  |  |  |  |  |
| Stroop dot time | -0.06 (-0.18, 0.06) | 0.32 |  | 0.04 (-0.06, 0.15) | 0.40 |  | 0.07 (-0.07, 0.20) | 0.33 |
| Stroop color time | -0.07 (-0.18, 0.04) | 0.19 |  | -0.04 (-0.13, 0.06) | 0.45 |  | 0.06 (-0.06, 0.18) | 0.32 |
| Stroop interference score | -0.07 (-0.18, 0.05) | 0.27 |  | -0.08 (-0.17, 0.02) | 0.11 |  | 0.00 (-0.12, 0.12) | 0.95 |

# **Supplement Table S4. The association between loneliness and cognitive function (NP battery tests)**

| **Neurocognitive test scores** | **Incident loneliness** | |  | **Transient loneliness** | |  | **Persistent loneliness** | |
| --- | --- | --- | --- | --- | --- | --- | --- | --- |
|  | *β* (95%CI) | P value |  | *β* (95%CI) | P value |  | *β* (95%CI) | P value |
| **Model 1 (n = 1924)** |  |  |  |  |  |  |  |  |
| LM - Delayed Recall | 0.06 (-0.11, 0.22) | 0.49 |  | 0.00 (-0.13, 0.13) | 0.99 |  | -0.01 (-0.17, 0.15) | 0.88 |
| LM - Recognition | 0.08 (-0.05, 0.22) | 0.22 |  | 0.03 (-0.09, 0.16) | 0.60 |  | -0.19 (-0.36, -0.03) | 0.02 |
| TMT A (revised z-score) † | -0.08 (-0.19, 0.04) | 0.18 |  | -0.08 (-0.18, 0.01) | 0.08 |  | -0.12 (-0.24, -0.01) | 0.04 |
| TMT B (revised z-score) † | -0.06 (-0.16, 0.04) | 0.24 |  | -0.08 (-0.16, 0.00) | 0.04 |  | -0.09 (-0.19, 0.01) | 0.08 |
| VR - Delayed Recall | 0.01 (-0.15, 0.17) | 0.89 |  | -0.11 (-0.22, 0.01) | 0.08 |  | -0.11 (-0.26, 0.05) | 0.19 |
| VR - Recognition | -0.04 (-0.19, 0.10) | 0.56 |  | 0.00 (-0.11, 0.11) | 0.99 |  | -0.20 (-0.35, -0.04) | 0.01 |
| Boston Naming Test | 0.03 (-0.11, 0.18) | 0.66 |  | 0.01 (-0.10, 0.12) | 0.90 |  | -0.02 (-0.17, 0.13) | 0.79 |
| **Model 2 (n = 1831)** |  |  |  |  |  |  |  |  |
| LM - Delayed Recall | 0.04 (-0.13, 0.21) | 0.64 |  | -0.02 (-0.15, 0.12) | 0.80 |  | -0.04 (-0.21, 0.14) | 0.69 |
| LM - Recognition | 0.07 (-0.07, 0.22) | 0.30 |  | 0.02 (-0.12, 0.15) | 0.80 |  | -0.22 (-0.40, -0.04) | 0.01 |
| TMT A (revised z-score) † | -0.03 (-0.15, 0.09) | 0.58 |  | -0.04 (-0.14, 0.06) | 0.40 |  | -0.05 (-0.18, 0.08) | 0.48 |
| TMT B (revised z-score) † | -0.05 (-0.16, 0.06) | 0.38 |  | -0.05 (-0.13, 0.03) | 0.21 |  | -0.04 (-0.15, 0.06) | 0.43 |
| VR - Delayed Recall | 0.04 (-0.12, 0.20) | 0.59 |  | -0.08 (-0.20, 0.05) | 0.22 |  | -0.07 (-0.23, 0.09) | 0.39 |
| VR - Recognition | 0.00 (-0.14, 0.15) | 0.96 |  | 0.04 (-0.07, 0.15) | 0.47 |  | -0.15 (-0.31, 0.01) | 0.06 |
| Boston Naming Test | 0.03 (-0.12, 0.18) | 0.70 |  | -0.01 (-0.13, 0.10) | 0.84 |  | -0.03 (-0.19, 0.13) | 0.71 |

# **Supplement Table S5. The associations between loneliness and longitudinal changes in cognitive function (Δ) stratified by sex and depression status.**

| **Cognitive function among different subgroups** | **Incident loneliness** | |  | **Transient loneliness** | |  | **Persistent loneliness** | |
| --- | --- | --- | --- | --- | --- | --- | --- | --- |
|  | *β* (95%CI) | P value |  | *β* (95%CI) | P value |  | *β* (95%CI) | P value |
| **Female (n = 1333)** |  |  |  |  |  |  |  |  |
| CERAD total score | -0.02 (-0.17, 0.12) | 0.74 |  | -0.03 (-0.17, 0.11) | 0.68 |  | -0.01 (-0.19, 0.17) | 0.91 |
| CERAD recall score | 0.03 (-0.09, 0.16) | 0.58 |  | -0.01 (-0.11, 0.10) | 0.91 |  | -0.05 (-0.19, 0.09) | 0.51 |
| CERAD retention score | 0.05 (-0.09, 0.18) | 0.49 |  | -0.04 (-0.16, 0.07) | 0.45 |  | -0.06 (-0.20, 0.09) | 0.42 |
| Stroop dot time | 0.02 (-0.11, 0.16) | 0.73 |  | 0.02 (-0.09, 0.13) | 0.75 |  | -0.04 (-0.18, 0.09) | 0.53 |
| Stroop color time | 0.09 (-0.06, 0.25) | 0.23 |  | -0.01 (-0.16, 0.13) | 0.87 |  | 0.00 (-0.16, 0.17) | 0.96 |
| Stroop interference score | 0.06 (-0.10, 0.21) | 0.48 |  | -0.05 (-0.19, 0.09) | 0.47 |  | 0.07 (-0.09, 0.22) | 0.39 |
| **Male (n = 1147)** |  |  |  |  |  |  |  |  |
| CERAD total score | 0.00 (-0.22, 0.22) | 0.99 |  | 0.02 (-0.19, 0.22) | 0.87 |  | 0.02 (-0.21, 0.26) | 0.84 |
| CERAD recall score | -0.01 (-0.22, 0.19) | 0.89 |  | -0.05 (-0.24, 0.14) | 0.59 |  | -0.30 (-0.56, -0.04) | 0.02 |
| CERAD retention score | 0.03 (-0.21, 0.27) | 0.78 |  | -0.05 (-0.24, 0.14) | 0.60 |  | -0.30 (-0.55, -0.06) | 0.01 |
| Stroop dot time | 0.02 (-0.14, 0.18) | 0.81 |  | -0.05 (-0.21, 0.11) | 0.58 |  | -0.12 (-0.30, 0.07) | 0.23 |
| Stroop color time | 0.17 (-0.07, 0.40) | 0.17 |  | 0.19 ( 0.04, 0.33) | 0.01 |  | 0.33 ( 0.09, 0.58) | 0.01 |
| Stroop interference score | 0.11 (-0.09, 0.31) | 0.28 |  | 0.14 (-0.02, 0.30) | 0.08 |  | 0.32 ( 0.09, 0.55) | 0.01 |
| **No depression** (n = 2278) |  |  |  |  |  |  |  |  |
| CERAD total score | -0.06 (-0.20, 0.08) | 0.41 |  | 0.01 (-0.11, 0.13) | 0.85 |  | -0.04 (-0.21, 0.13) | 0.67 |
| CERAD recall score | 0.02 (-0.10, 0.13) | 0.79 |  | -0.03 (-0.13, 0.06) | 0.53 |  | -0.13 (-0.29, 0.04) | 0.12 |
| CERAD retention score | 0.04 (-0.09, 0.17) | 0.52 |  | -0.06 (-0.17, 0.04) | 0.24 |  | -0.14 (-0.30, 0.02) | 0.09 |
| Stroop dot time | -0.01 (-0.13, 0.11) | 0.83 |  | 0.00 (-0.10, 0.09) | 0.95 |  | -0.18 (-0.32, -0.03) | 0.02 |
| Stroop color time | 0.14 ( 0.00, 0.29) | 0.06 |  | 0.07 (-0.04, 0.17) | 0.22 |  | 0.11 (-0.07, 0.29) | 0.23 |
| Stroop interference score | 0.12 (-0.03, 0.26) | 0.11 |  | 0.01 (-0.09, 0.12) | 0.79 |  | 0.21 ( 0.03, 0.39) | 0.02 |
| **Depression** (n = 202) |  |  |  |  |  |  |  |  |
| CERAD total score | 0.05 (-0.31, 0.41) | 0.80 |  | -0.36 (-0.83, 0.11) | 0.14 |  | 0.01 (-0.35, 0.37) | 0.97 |
| CERAD recall score | -0.04 (-0.40, 0.31) | 0.82 |  | -0.11 (-0.60, 0.37) | 0.65 |  | -0.20 (-0.57, 0.17) | 0.30 |
| CERAD retention score | 0.03 (-0.37, 0.44) | 0.87 |  | 0.03 (-0.48, 0.54) | 0.91 |  | -0.17 (-0.56, 0.21) | 0.38 |
| Stroop dot time | 0.26 (-0.08, 0.59) | 0.14 |  | 0.02 (-0.42, 0.46) | 0.93 |  | 0.19 (-0.14, 0.51) | 0.26 |
| Stroop color time | 0.28 (-0.30, 0.86) | 0.34 |  | 0.26 (-0.42, 0.93) | 0.45 |  | 0.24 (-0.34, 0.81) | 0.42 |
| Stroop interference score | 0.05 (-0.43, 0.54) | 0.83 |  | 0.18 (-0.38, 0.74) | 0.52 |  | 0.13 (-0.34, 0.61) | 0.58 |

# **Supplement Table S6. The associations between loneliness and AD8 and MoCA scores stratified by sex and depression status**

| **Neurocognitive scores** | **Incident loneliness** | |  | **Transient loneliness)** | |  | **Persistent loneliness** | |
| --- | --- | --- | --- | --- | --- | --- | --- | --- |
|  | β (95%CI) | P value |  | β (95%CI) | P value |  | β (95%CI) | P value |
| **All subjects** (n = 2461) |  |  |  |  |  |  |  |  |
| AD8 score | 0.18 ( 0.07, 0.30) | 0.002 |  | 0.15 ( 0.06, 0.24) | 0.002 |  | 0.46 ( 0.31, 0.61) | <0.001 |
| MoCA score | -0.01 (-0.15, 0.12) | 0.83 |  | -0.04 (-0.15, 0.06) | 0.43 |  | -0.10 (-0.24, 0.03) | 0.14 |
| **Female** (n = 1332) |  |  |  |  |  |  |  |  |
| AD8 score | 0.13 (-0.02, 0.28) | 0.09 |  | 0.07 (-0.04, 0.18) | 0.23 |  | 0.45 ( 0.25, 0.65) | <0.001 |
| MoCA score | -0.08 (-0.24, 0.08) | 0.31 |  | -0.06 (-0.20, 0.08) | 0.37 |  | -0.17 (-0.36, 0.02) | 0.08 |
| **Male** (n = 1139) |  |  |  |  |  |  |  |  |
| AD8 score | 0.27 ( 0.09, 0.46) | 0.004 |  | 0.29 ( 0.12, 0.45) | <0.001 |  | 0.49 ( 0.25, 0.72) | <0.001 |
| MoCA score | 0.10 (-0.14, 0.33) | 0.43 |  | -0.02 (-0.18, 0.15) | 0.82 |  | -0.02 (-0.21, 0.18) | 0.87 |
| **No depression** (n = 2261) |  |  |  |  |  |  |  |  |
| AD8 score | 0.10 (-0.01, 0.21) | 0.08 |  | 0.13 ( 0.04, 0.22) | 0.004 |  | 0.27 ( 0.12, 0.42) | <0.001 |
| MoCA score | -0.01 (-0.17, 0.15) | 0.91 |  | -0.05 (-0.16, 0.06) | 0.35 |  | -0.06 (-0.22, 0.11) | 0.50 |
| **Depression** (n = 200) |  |  |  |  |  |  |  |  |
| AD8 score | 0.30 (-0.20, 0.80) | 0.24 |  | 0.35 (-0.32, 1.01) | 0.31 |  | 0.59 ( 0.10, 1.09) | 0.02 |
| MoCA score | -0.03 (-0.46, 0.40) | 0.9 |  | 0.12 (-0.45, 0.68) | 0.69 |  | -0.14 (-0.57, 0.29) | 0.52 |

# **Supplementary Figure S1. The dose-response relationship between cumulative loneliness and AD8 Score and MoCA score.**

**
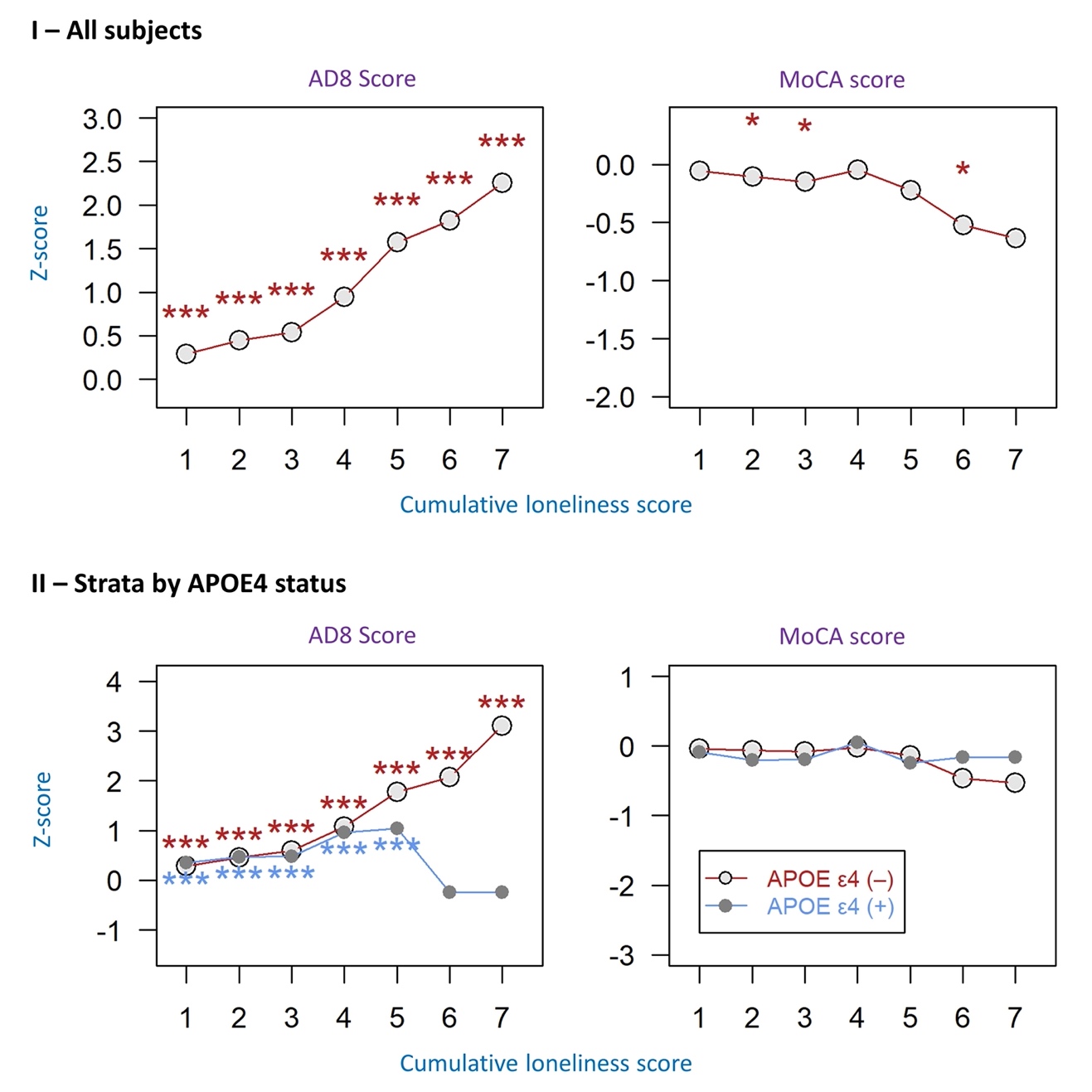
**

# **Supplementary Figure S2. Directed Acyclic Graph.**


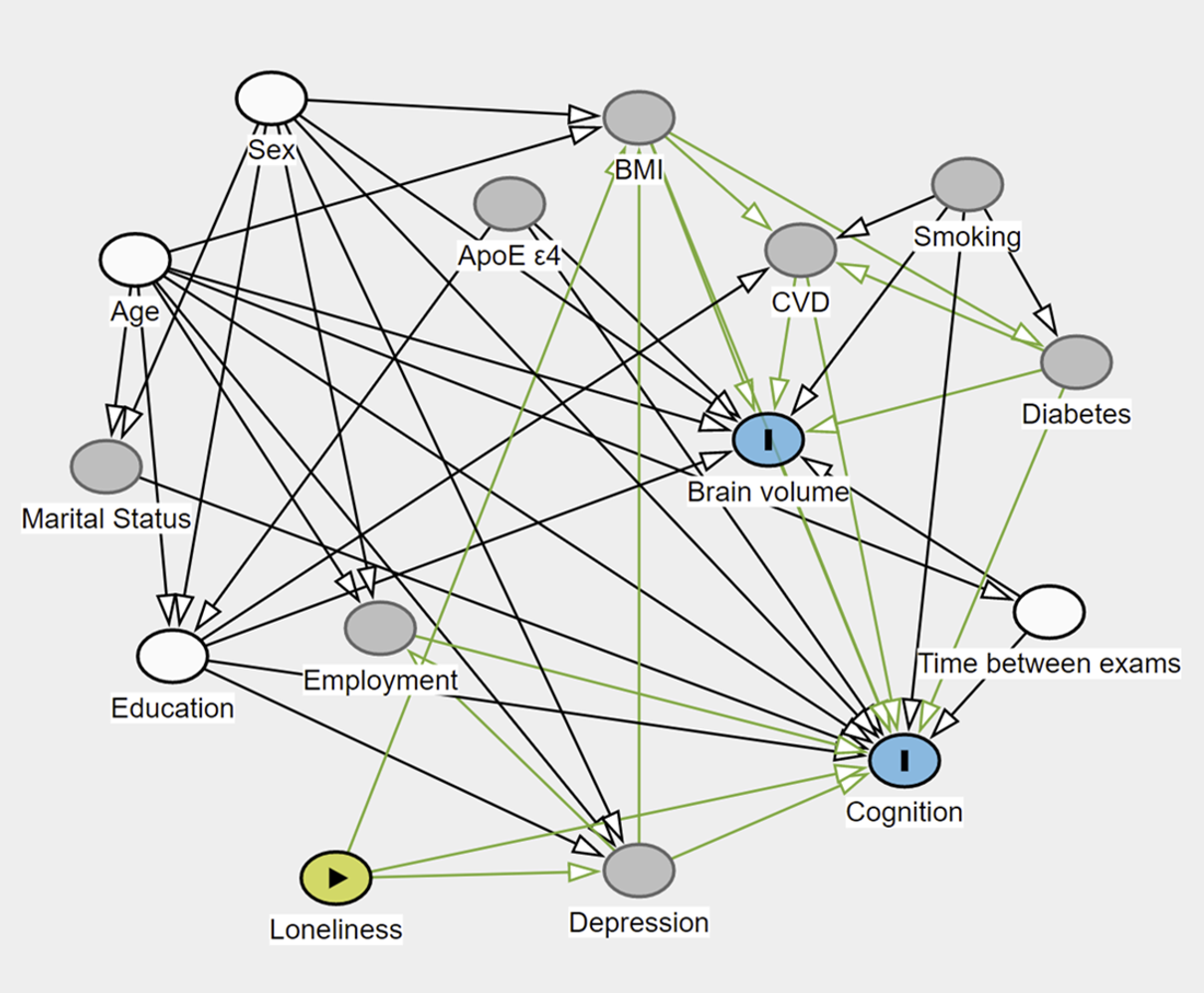

Supplement: Supplementary file 2 [file mmc2.docx]
